# The Prion 2018 round tables (II): Aβ, tau, α-synuclein… are they prions, prion-like proteins, or what?

**DOI:** 10.1080/19336896.2019.1569451

**Published:** 2019-01-15

**Authors:** Hasier Eraña

**Affiliations:** Atlas Molecular Pharma, Parque Tecnológico de Bizkaia, Derio, Spain; CIC bioGUNE, Parque Tecnológico de Bizkaia, Derio, Spain

**Keywords:** Prion, Transmissible Spongiform Encephalopathy, prion-like, prionoid, neurodegeneration

## Abstract

The description of prions as causal agents of Transmissible Spongiform Encephalopathies (TSE), is nowadays accepted as an important breakthrough in biology as revealed the existence of a completely new group of pathogens and a new way of transmission for biological information. A common feature of many neurodegenerative disorders is the presence of protein aggregates in the nervous system and as evidences highlighting the similarities of these proteins with TSE-causing prions increase, the line separating the infectious prions from other protein aggregates becomes thinner than previously thought. However, instead of encompassing all these amyloidogenic proteins under the umbrella term "prion", new terminology has raised including the terms prion-like, prionoid, quasi-prion or propagon.

The International Prion Conference held in Santiago de Compostela in 2018, offered the perfect forum to discuss this topic and maybe set the basis for an agreed terminology. For that, a round table was organized with several experts on the field to discuss whether AÎ², tau, Î±-synuclein and others are prions, prion-like proteins, or should be named otherwise. This commentary intends to summarize the topics discussed at the round table and shed some light on this controverted topic, drawing together the opinions of many experts participating at the session.

More than three decades have passed since the causal agents of transmissible spongiform encephalopathies (TSEs) were named prions [1], taking the biological science community by storm. The description of the first proteinaceous pathogen completely devoid of nucleic acids, implicated the existence of a completely novel biological mechanism for information transmission based on protein conformation, although its acceptance by the scientific community required few years and an overwhelming amount of evidences. In 1982, Stanley Prusiner defined prions as proteinaceous infectious particles and proposed the existence of an alternative propagation mechanism and thus, a completely new group of pathogens. Initially heretical, his discovery was actually an important breakthrough in biology and nowadays it seems obvious that TSE-causing prions were just the tip of the iceberg.

**Figure 1.**
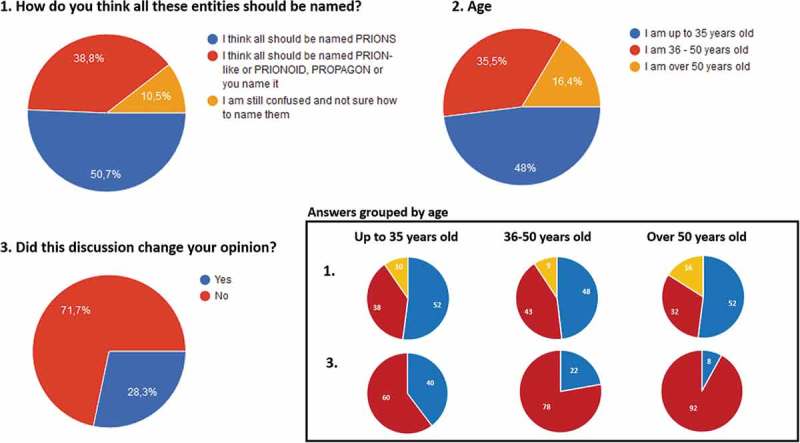
Results from the poll done during the round table. 152 answers were collected from the audience that are represented as pie charts in percentages.

Over the years, as researchers in the field of TSEs gathered enough evidence demonstrating unequivocally the proteic nature of prions, a similar phenomenon was discovered in yeast. In 1994, Reed Wickner described a particular phenotype in yeast that seemed to be based on a conformational rearrangement of a normal host protein that was catalyzed by the misfolded form itself and transmitted from cell to cell [2]. Thus, he defined this non-Mendelian proteic element as a yeast prion, due to the same propagation and transmission mechanism. Since then, the yeast prion family has been steadily growing and many other self-perpetuating protein conformers have been found, including some involved in distinct pathologies. The notable growth of age-related neurodegenerative disorders linked to increased life expectancy has boosted considerably investigations to determine their pathogenesis. The intensive research performed during the last two decades has shown that a common feature of many neurodegenerative disorders is the presence of aggregates of misfolded endogenous proteins in specific regions of the nervous system. This is the case of Parkinson’s disease, Multiple System Atrophy and other synucleinopathies, Alzheimer’s disease, Frontotemporal Dementia and other tauopathies, among others. As evidences highlighting the similarities of these proteins with TSE-causing prions increase [3], the line separating the infectious prions from the non-infectious and functional amyloids or pathologic aggregates becomes thinner than previously thought. However, instead of encompassing all these amyloidogenic proteins under the umbrella term ‘prion’, as it was done readily for those found in yeast, a new and in occasions confusing terminology has raised including the terms prion-like, prionoid, quasi-prion or propagon [3–5], mainly because certain characteristics of TSE-causing prions have not been demonstrated yet for all these proteins.

Thus, the International Prion Conference held in Santiago de Compostela in 2018, with most of the researchers working on TSE-causing prions, yeast prions and many colleagues dealing with other amyloidogenic proteins from a prion viewpoint, seemed the perfect forum to discuss the topic and maybe set the basis for an agreed terminology. For that, a round table was organized to discuss whether Aβ, tau, α-synuclein and others are prions, prion-like proteins, or how should be named all these entities with a behavior clearly reminiscent of prions. The round table brought together a great panel of researchers: Dr. Corinne Lasmézas from The Scripps Research Institute, Dr. Claudio Soto from The University of Texas Medical School at Houston, Nobel laureate Dr. Stanley Prusiner from the University of California San Francisco and Dr. Erdem Tamgüney from the German Center for Neurodegenerative Diseases, who introduced and discussed the matter under the moderation of Dr. Mathias Jucker from the German Center for Neurodegenerative Diseases and Hertie Institute of the University of Tübingen, with the assistance of Dr. Hasier Eraña from ATLAS molecular pharma/CIC bioGUNE. The debate was also open to all the attendees, many of whom participated actively providing their opinions, making it a lively discussion where different points of view were represented. Moreover, an on-line survey was done live during the session to collect the general opinion of the attendees on the subject and determine if this kind of debate could convince people to change their minds. This small summary of the round table intends to compile some of the debated topics and withdraw few conclusions from an intense session that lasted almost two hours.

Each of the participants made a brief introduction presenting the latest evidences of other proteins showing prion behavior and about the differences observed between TSE-causing prions and other pathogenic proteins. Dr. Prusiner already stated his position in his Opening lecture of the conference, ‘Unified view of Prion biology and diseases’. For the sake of clarity, the distinct viewpoints that emerged from the posterior debate have been considered in separate currents of opinion.

The initial position of some people on this topic is that semantics are not so important after all. Is it really pivotal to use a unified terminology? As long as the molecular events are described in detail and research is performed using all the possible approaches, is a rigorous classification necessary to keep advancing on our knowledge about all these pathogenic proteins? Certainly, calling them prions, prion-like agents, prionoids or pathogenic proteins, does not change their nature and their biological properties. However, an agreed terminology based on the increasingly convincing evidences of mechanistic similarities between the proteins causing distinct neurodegenerative diseases could be of upmost importance for the development of therapies and diagnostic methods. This shared terminology may finally convince experts working on protein misfolding related neurodegenerative disorders to adopt methodologies well-known in the field of TSE research, boosting new therapeutic strategies against these devastating disorders. Yeast prions, despite their differences with TSE-causing prions, have become a valuable model to study the properties of proteins that can undergo a conformational change to self-perpetuating isoforms and have contributed to our knowledge on the events of misfolding, strain variability and propagation of the aggregates, among others. All these could have probably been achieved without a common definition, although it may have taken much longer to establish the relation between the functional amyloids found in yeast and the pathogenic prions found in mammals. Therefore, grouping these entities under the definition of prion favored research in both areas, TSEs and the non-Mendelian proteic information transmission elements from yeast.

Those defending the use of names alternative to prion for the proteins behind other neurodegenerative diseases or for the functional amyloids found in diverse organisms, such as prion-like, prionoid or quasi-prion, argue that these proteins share several properties with prions but not all of them such as infectivity or inter-individual transmissibility. Several researchers on the field use the term prion exclusively for TSE-causing prions and yeast prions based on the original definition of the term coined by Stanley Prusiner in 1982, ‘proteinaceous infectious particles that resist inactivation by procedures that modify nucleic acids’ [1]. In this definition, infectious could be understood as the ability of an agent to invade and multiply in body tissues or the ability to be passed from an individual to another. Thus, the discovery of yeast prions in 1994, able to be transmitted from cell-to-cell, fulfilled the requirements to be termed prions. However, yeast prions were not precisely pathogenic and therefore they fit in a definition of infectious that does not involve disease, although these two words are frequently associated erroneously [6]. The discovery of new pathogenic and also functional self-perpetuating misfolded proteins in multicellular organisms that behaved in many ways like TSE-causing prions, led some researchers to adopt a broader definition of prion. As proposed by Stanley Prusiner in 2013, prions could be considered ‘proteins that acquire alternative conformations and become self-propagating’ [7]; or by Lary Walker and Mathias Jucker ‘proteinaceous nucleating (instead of infectious) particles’ [8]. Still, many researchers in the field of TSEs and others studying other neurodegenerative diseases associated with protein misfolding stick to the original definition and defend the need of new terminology. Different proposals have been published, but the terms that are used most when referring to these disorders are ‘prion-like’ and rarely also ‘prionoid’ and ‘quasi-prion’.

Some people thus may argue that prions would be exclusively TSE-causing prions, some yeast prions, α-synuclein prions causing Multiple System Atrophy (MSA) and the systemic A-amyloidosis in cheetah, for which inter-individual transmissibility has been unequivocally demonstrated. The rest of the self-propagating misfolded proteins should be considered for the time being prion-like entities that encompasses quasi-prions and prionoids. Quasi-prions would be those protein aggregates for which horizontal transmissibility has never been shown although they are able to be passed to progeny, such as some yeast proteins and the bacterial RepA protein [4]. Finally, prionoids have been defined as ‘protein aggregates that can propagate and spread between cells but for which transmissibility between individuals has not yet been demonstrated’ [9]. As useful as this terminology can be nowadays to distinguish between different self-propagating protein aggregates it is most likely a temporary solution, and as it happened with MSA-causing prions, many other aggregates will need to be relocated as the right ways for inter-individual transmission are found. In addition, a classification based on inter-individual transmissibility or infectivity is *per se* complicated due to the poor definition of such concepts. Clearly, transmissibility depends not only on the characteristics of the protein aggregate, but also on the susceptibility of the new host and on finding the right sources of administration. For example, Gerstmann-Sträussler-Scheinker syndrome-causing prions are poorly transmissible, if transmissible at all, unless the right models are used. Moreover, cell-to-cell transmissibility and spreading between tissues have already been proved for most of these aggregates and they could be considered intrinsic properties of such kind of entities able to self-perpetuate, leading to the idea that they could do the same between individuals if able to reach the right propagation sites. Therefore, are TSE-causing prions unique in their ability of transmission through natural sources, rather than in their inter-individual transmissibility? But is this true for all the TSE-causing prions? Although the classification of self-propagating misfolded proteins could be convenient and useful, there are still some issues to clarify regarding transmissibility and infectivity, that should be addressed before a proper classification can be established. Similarly, other classifications could be proposed prior to the definitive demonstration of interindividual transmissibility. For instance, prions could be those composed of prion protein, yeast prions those self-propagating proteins found in fungae and prion-like could encompass all other proteins able to misfold, induce aberrant conformation to endogenous proteins and propagate from cell-to-cell.

Finally, the viewpoint of researchers defending that all these proteins should be called prions needs to be considered. There is a growing body of evidence describing features thought to be unique for TSE-causing prions, for proteins implicated in other neurodegenerative diseases. For most of them, self-propagating capacity has been shown *in vitro*, in cultured cells or even in animal models, as well as cell-to-cell spreading ability. Thus, most of them comply with the requirements to be called prions based on the broader definition of ‘proteins that acquire alternative conformations and become self-propagating’. However, the infectivity, understood as serial inter-individual transmissibility is yet to proof for some of these aggregates in models not bearing mutations related to disease susceptibility (E.g. homogenates of Parkinson’s diseases and Dementia with Lewy bodies are unable to induce pathology unless a mutated human α-synuclein transgene is expressed in homozygosis). This fact has actually become the strongest argument not to categorize them as *bona fide* prions. Similarly, neurotoxicity mechanisms are still unknown or poorly characterized in many cases and may differ from each other since different proteins are involved in each disease. Nonetheless, similarities regarding the molecular basis of protein misfolding and propagation are obvious and even complex phenomena such as conformational strain diversity has been observed for many of the proteins under discussion. In the light of these similarities, many researchers stand for including all these proteins under the term prion, as it was originally done for yeast prions based on the molecular mechanisms, despite the differences between functional amyloids and infectious protein pathogens. Similar to viruses, which includes strikingly different entities, the idea would be to name all the misfolded and self-propagating proteins, prions. Apart from the gathered evidences for shared mechanisms, a strong argument was formulated during the debate by Dr. Soto making use of the abductive reasoning, ‘If it looks like a duck, swims like a duck, and quacks like a duck, then it probably is a duck’. The main objection to this idea, putting aside scientific uncertainties as inter-individual transmissibility, is due to the impact that this terminology could have on the public opinion, the clinical practice and research. Prions, being the causal agents of TSEs are synonyms of the mad cow epidemics in the public opinion, therefore calling prion diseases two of the most prevalent neurodegenerative diseases such as Alzheimer’s or Parkinson’s diseases would set alarm bells ringing, in spite of the low or almost null risk of natural transmission. Moreover, if these proteins are defined as prions and thus, the possibility of inter-individual transmission assumed, their handling would require specific biosafety conditions with unpredictable repercussion in research, clinical practice and patient care. Therefore, the precautionary principle should be applied before definitively associating all the misfolding, self-propagating proteins with prions, with all their bad press, and maybe increase our efforts on scientific dissemination before any decision is taken.

What the round table allowed to conclude clearly is that there are divergent opinions even among the researchers on TSE-causing prions, on yeast prions and those that study other protein misfolding-related disorders from a prion, prionoid or prion-like viewpoint. In fact, this was also reflected by the poll done during the session, which brought up three simple questions to the audience and for which 152 answers were collected (Figure 1). About 50% of the audience thinks all these entities should be named prions, while almost 40% considers we should use new terminology and around 10% feels confused and is not sure about how they should be named. Therefore, the debate remains open and there is plenty to discuss before a consensus is reached, although the rapidly increasing amount of detailed information on the biology of all these proteins may solve the debate for us. Meanwhile, researchers in the area of TSEs and other protein misfolding-related neurodegenerative disorders need to properly define some terms we use daily, because in my humble opinion, scientific discussion cannot depend on individual considerations or beliefs. And until we can fill all the gaps in our knowledge about these proteic entities, we may need to anticipate and clarify terminology issues to establish a common work frame that would benefit all the researchers dealing with these proteins.

## Aknowledgements

The author would like to thank all the participants at the Round table for their contribution to the debate as well as to those on the audience that expressed their viewpoint through comments or through their participation at the poll. Specially, the author would like to aknowledge Joaquín Castilla, Jesús R. Requena, Claudio Soto, Corinne Lasmézas and Mathias Jucker for their useful advice on this Round table summary.
